# Editorial: Myelopathies and spinal cord injuries: advances and controversies in pathophysiology, diagnosis, and treatment

**DOI:** 10.3389/fneur.2024.1468613

**Published:** 2024-09-03

**Authors:** Michael Poon, Miltiadis Georgiopoulos, Oliver Lasry, Colin Chalk, Andreas K. Demetriades

**Affiliations:** ^1^Centre for Clinical Brain Sciences, University of Edinburgh, Edinburgh, United Kingdom; ^2^Department of Neurosurgery, Royal Infirmary of Edinburgh, Edinburgh, United Kingdom; ^3^Edinburgh Spinal Surgery Outcome Studies Group, Edinburgh, United Kingdom; ^4^Spinal Surgical Unit, Trauma and Orthopaedics, Swansea Bay University Health Board, Swansea, United Kingdom; ^5^Department of Neurology and Neurosurgery, McGill University, Montreal, QC, Canada

**Keywords:** myelopathy, spinal cord injury, pathophysiology, spinal surgery, spinal cord surgery, controversies

Spinal cord injury (SCI) represents a temporary or permanent change to neurological function. Presentation is variable owing to the mechanism and severity of injury. Undoubtedly there have been improvements in pre-hospital care, diagnostics, surgical options, and intensive care therapies for people with SCI. However, the burden of SCIs on population health remains high due to their propensity to cause long-term disability, lower quality of life, reduced life expectancy, and high healthcare costs (1). Despite an increasing understanding of the biological processes underpinning SCI (2, 3), there is no universally effective treatment. Incremental and meaningful improvement of patient outcomes following SCI requires a multifaceted approach. Advances in neuro-regeneration provide hope for novel therapies that enhance functional recovery. In the complex context of SCI, articles in this Research Topic address different aspects of SCI—from diagnosis to future therapies.

Transverse myelitis is a rare inflammatory disorder that causes SCI. Evidence demonstrated that neurological disorders can complicate COVID-19 (4). Kawama et al. reported three patients diagnosed with myelitis associated with SARS-CoV-2 infection without radiographic abnormality on T2-weight MRI imaging, which is usually a feature of myelitis (5). Electrophysiological studies were helping in spinal cord localization to enable prompt immunosuppressive therapies. Despite the latency period between SARS-CoV-2 infection and neurological manifestation seen in the reported cases and those from the literature, Kawama et al. showed that patients can make good neurological recovery.

Primary and secondary injuries associated with traumatic SCI determine the neurological outcomes. Pharmacological neuroprotective agents may enhance recovery after traumatic SCI (6). To facilitate further investigations into the efficacy of neuroprotective agents, biomarkers for SCI severity may aid the stratification of SCI patients for trials. Li et al. set out to identify blood biomarkers associated with SCI severity. They first used transcriptomic data from a biorepository of people with SCI and healthy controls to identify a set of differentially expressed immune-related genes. Using functional enrichment analysis, co-expression network analysis, and immune deconvolution methods, the authors reported immune characteristics correlating with SCI severity according to the American Spinal Injury Association (ASIA) impairment scale. The validation experiments using quantitative polymerase chain reaction supported their findings in 10 external SCI patients. Their findings raise the possibility of using peripheral blood biomarkers to quantify the neuroinflammation following SCI. Solou et al. presented a literature review of six observational studies reporting surgical management of adults with SCI but without spinal canal compromise. Surgical management unsurprisingly varied; groups included laminectomy, laminoplasty, and laminectomy plus duroplasty or durotomy. Recommendations cannot be taken from this narrative review though consideration of intra-spinal pressure may guide surgical options. Whether the act of opening the theca changes the neuroinflammatory process after SCI may be worth investigating.

Regenerative neurology holds promises for the treatment of SCI. Xia et al. summarized how mesenchymal stem cells (MSCs) may form a treatment for SCI due to their roles in reducing inflammation, promoting axonal regeneration, and vascular repair. The authors reviewed the relevant mechanistic pathways and provided an overview of the clinical applications in studies. As highlighted by the authors, there are many challenges to overcome in ensuring the survival of transplanted MSCs and inducing the intended effects. Further experimental studies to determine the optimal conditions for MSCs in combination with *in vivo* imaging (7) will get us closer to successful later phase clinical trials.

Degenerative cervical myelopathy (DCM) is a prevalent cause of SCIs in adults. Surgical decompression is a management option, but the treatment response is variable. There is emerging evidence that cervical muscle morphology, which can be assessed on routine imaging, may be associated with functional outcomes after surgery for DCM. Naghdi et al. measured cervical muscle morphology in 171 patients undergoing surgery for DCM. Of the morphometric measures they collected, greater asymmetry and fatty infiltration were associated with worse outcomes at 6 and 12 months. This finding is not surprising since muscle morphology is related to surgical outcomes in degenerative lumbar spinal disease (8). While findings by Naghdi et al. may aid patient selection, it is important to consider whether this is a modifiable factor or a proxy measure for frailty.

Central cord syndrome (CCS) is a form of SCI characterized by prominent upper limb dysfunction with relative sparing of lower limb function. Advances in our understanding of the spinal cord prompted (Shakil et al.) to discuss the pathophysiology of CCS. They highlighted recent evidence from studies on humans and primates that supports the lack of somatotopic representation of the corticospinal tract in the spinal cord (9). Injury associated with CCS is a diffuse process. The authors also discussed the clinical application of this understanding in the context of two illustrative cases.

Taken together, the articles in this Research Topic offer a wide perspective on SCI and serve to inform the readers of developments in our understanding of the pathophysiology and clinical management of SCI.
